# Investigation of antimicrobial resistance and antimicrobial resistance genes in *Staphylococcus aureus* and coagulase-negative staphylococci isolated from rabbit

**DOI:** 10.14202/vetworld.2024.1328-1335

**Published:** 2024-06-21

**Authors:** Nawarat Suriyakhun, Arunee Jangsangthong, Witawat Tunyong, Thida Kong-Ngoen, Sirijan Santajit, Nitaya Indrawattana, Shutipen Buranasinsup

**Affiliations:** 1 Prasu-Arthorn Animal Hospital, Faculty of Veterinary Science, Mahidol University, 73170 Nakhon Pathom, Thailand; 2 Department of Pre-clinic and Applied Animal Science, Faculty of Veterinary Science, Mahidol University, 73710 Nakorn Pathom, Thailand; 3 Department of Microbiology and Immunology, Faculty of Tropical Medicine, Mahidol University, 10400 Bangkok, Thailand; 4 Department of Medical Technology, School of Allied Health Sciences, Walailak University, 80160 Nakhon Si Thammarat, Thailand; 5 Research Center in Tropical Pathobiology, Walailak University, 80160 Nakhon Si Thammarat, Thailand; 6 Siriraj Center of Research and Excellence in Allergy and Immunology, Faculty of Medicine Siriraj Hospital, Mahidol University, 10700 Bangkok, Thailand

**Keywords:** antimicrobial resistance, antimicrobial resistance genes, coagulase-negative staphylococci, rabbit, *Staphylococcus aureus*

## Abstract

**Background and Aim:**

Staphylococci, which inhabit skin and mucous membranes in humans and animals, are opportunistic pathogens. Coagulase-positive and coagulase-negative staphylococci (CoNS) are the two main groups. Clinical abscesses in rabbits often harbor *Staphylococcus aureus* and CoNS. This study estimated *S. aureus* and CoNS prevalence, resistance profiles, antimicrobial-resistant genes, and the accessory gene regulator (*agr*) group in rabbit clinical abscesses.

**Materials and Methods:**

Sixty-seven abscesses were gathered from 67 rabbits who visited Prasu-Arthorn Animal Hospital in Nakornpathom, Thailand, from January 2014 to October 2015. Thirty-four subcutaneous, 29 dental, 2 ocular, 1 mammary gland, and 1 uterine abscess were present. Conventional methods, including Gram staining, mannitol fermentation, hemolysis on blood agar, catalase testing, and coagulase production, identified and isolated *S. aureus* and CoNS from all abscesses. All *S. aureus* and CoNS isolates underwent antimicrobial susceptibility testing using the disk diffusion method. Polymerase chain reaction was used to detect the presence of *bla*Z, *aac*A-*aph*D, *msr*A, *tet*K, *gyr*A, *grl*A, *dfr*G, and *cfr* antimicrobial-resistant genes. Methicillin resistance was identified through the detection of a cefoxitin-resistant phenotype and the presence of *mec*A gene. Further investigation was conducted on the *agr* group of *S. aureus* isolates.

**Results:**

In 67 abscesses, we found 19 *S. aureus* isolates in 9 abscesses (13.43%) and 37 CoNS isolates in 18 abscesses (26.87%), both majorly located at subcutaneous sites. About 59.46% of CoNS isolates were methicillin-resistant compared to 5.26% of *S. aureus* isolates. Methicillin-resistant *S. aureus* (MRSA) and methicillin-resistant CoNS (MRCoNS) both displayed multidrug resistance (MDR). Both MRSA and MRCoNS expressed multiple antimicrobial resistance genes, including *bla*Z, *aac*A-*aph*D, *gyr*A, *grl*A, *msr*A, *tet*K, and *dfr*G. Approximately 73.68% of the *agr* groups were *agr* I, 15.79% were *agr* III, and 10.53% were *agr* II.

**Conclusion:**

This study found a high prevalence of MRCoNS with antimicrobial resistance and multiple antimicrobial-resistant genes in rabbits with clinical abscesses. The effectiveness of antibiotics against infections caused by such strains is a matter of concern. Owners should be educated about the importance of good hygiene practices and judicious antibiotic use to prevent widespread antimicrobial resistance.

## Introduction

Staphylococci inhabit the upper respiratory tract of humans and animals as normal flora on the skin and mucous membranes. These bacteria can adapt and thrive in the environment under adverse conditions, originating from it. Coagulase-positive staphylococci (CoPS) and coagulase-negative staphylococci (CoNS) are the two main groups [1, 2]. *Staphylococcus aureus*, a globally present zoonotic pathogen under CoPS, notoriously known for its broad virulence factors and antibiotic resistance, causes infections of high morbidity and mortality. In humans, *S. aureus* can cause a spectrum of diseases, from minor skin infections to severe conditions such as endovascular infections, pneumonia, septic arthritis, endocarditis, osteomyelitis, and sepsis [3]. Animals serve as significant reservoirs for human infections with *S. aureus* [4]. CoNS are commensal bacteria in humans and several animal species. These opportunistic pathogens pose substantial risks in health-care facilities, particularly through the application of medical devices. In small animal medicine, research on CoNS is less prevalent than that of *S. aureus*. Although isolated from different infection sites like the skin, ear canal, and respiratory tract, the complete virulence potential of CoNS remains uncertain due to their ability to rapidly acquire multiple resistance traits leading to multidrug resistance (MDR) against various antimicrobials [5-7]. *Staphylococcus epidermidis* and other species can make infections related to implanted medical devices particularly difficult to treat due to their ability to form biofilms. Despite extensive research on multidrug-resistant CoNS in humans, there is a paucity of data on this topic within veterinary medicine [8]. Staphylococci predominantly infect small dermal lesions in rabbits, making them a significant host for these bacteria. These infections have the potential to penetrate the subcutaneous tissue, leading to conditions such as pododermatitis, subcutaneous abscesses, and mastitis. Internal organ abscesses, including those of the lungs, liver, and uterus, can occasionally form. In severe cases, rabbit infections can lead to infertility, adverse production outcomes, and mortality [3, 9]. Staphylococci infections present a major concern due to the high prevalence of antibiotic resistance in these microbes. Methicillin-resistant staphylococci hold significant clinical importance. Staphylococci are also widely resistant to other antibiotics. They exhibit near-universal resistance to β-lactam antibiotics, such as penicillin (P) and its derivatives, which can be inactivated by β-lactamases. They can exhibit resistance to almost all antibiotics in a combined form [10]. Studies have consistently reported high levels of antimicrobial resistance in staphylococci obtained from companion animals [11–14]. Limited information is available on the antimicrobial resistance of staphylococci found in rabbits [9, 11].

The pathogenicity of *S. aureus* depends on the emergence of diverse accessory genes for cell wall-related and extracellular proteins. Genes responsible for virulence factors, secretory exotoxins, and hemolysins significantly impact *S. aureus*’s virulence and antimicrobial resistance. The accessory gene regulator (*agr)* locus, consisting of the genes *hld*, *agr*B, *agr*D, *agr*C, and *agr*A, manages the gene expression. The grouping of *agr* I, II, III, and IV is determined by distinct polymorphisms in *agr*D and *agr*C [15]. *Agr* groups have lately been linked to specific clinical conditions [16–22]. For instance, community-acquired methicillin-resistant *S. aureus* (MRSA) and isolates producing *S. aureus* toxic shock syndrome toxin-1 typically belonged to *agr* III. Meanwhile, glycopeptide–intermediately resistant *S. aureus* (GISA) isolates and exfoliatin-producing strains are more prevalent among isolates in *agr* II and IV, respectively [16]. The preponderance of clinical isolates is found to be comprised of *agr* I strains [15, 17–24].

This study estimated the prevalence of *S. aureus* and CoNS in rabbits, determined the antimicrobial resistance of these isolates, identified resistant genes, and classified *S. aureus* strains according to their *agr* group.

## Materials and Methods

### Ethical approval

Sample collection was conducted by an experienced veterinarian, following the guidelines of “Guide for the Care and Use of Laboratory Animals” [25]. All experimental procedures involving animals were approved by The Faculty of Veterinary Science-Animal Care and Use Committee, Mahidol University (protocol number MUVS-2013-35 and MUVS-2023-07-42).

### Study period and location

The study was conducted from January 2014 to October 2015. Samples were collected at Prasu-Arthorn Animal Hospital, Faculty of Veterinary Science, Mahidol University, Nakhonpathom Province.

### Specimen collection and bacterial isolation

Samples were obtained from 67 rabbits presenting with clinical abscesses at Prasu-Arthorn Animal Hospital in Nakornpathom Province. The owners granted permission for sample collection. Sixty-seven specimens were gathered, including 34 subcutaneous abscesses, 29 dental abscesses, 2 ocular abscesses, 1 mammary gland abscess, and 1 uterine abscess. Within 8 h of collection, all specimens were transferred to the Department of Veterinary Science, Mahidol University, in an Amies transport medium (Oxoid, UK). Three yellow colonies per specimen were grown on mannitol salt agar (Oxoid) at 37°C for 24–48 h and subsequently transferred to blood agar (Oxoid). The colonies were identified based on their hemolysis patterns using classical biochemical techniques, including Gram staining (Merck, Germany), catalase production (Merck), coagulation (Oxoid), and latex agglutination (Dryspot Staphytect plus, Oxoid) for protein A detection.

### Antimicrobial susceptibility testing

Antimicrobial susceptibility testing and interpretation were conducted using the disk diffusion method in accordance with the Clinical and Laboratory Standards Institute (CLSI) guidelines [26]. A total of 13 antimicrobial drugs were tested, including amikacin (30 μg), azithromycin (AZM) (15 μg), cefazolin (30 μg), cefoxitin (FOX) (30 μg), ceftriaxone (30 μg), chloramphenicol (C) (30 μg), ciprofloxacin (5 μg), doxycycline (DO) (30 μg), gentamicin (10 μg), moxifloxacin (MXF) (5 μg), norfloxacin (NOR) (10 μg), P (10 units), and trimethoprim/sulfamethoxazole (1.25/23.75 μg). *S. aureus* ATTC® 25923 (Manassas, USA) served as the control strain. Inhibition zones were measured and categorized according to the CLSI guidelines as susceptible (S), intermediate (I), or resistant (R) [26]. Methicillin-resistant strains were confirmed by the FOX-resistant phenotype.

### Detection of antimicrobial resistance and *agr* genes

The DNA extraction kit (Geneaid, Taiwan) was used to extract bacterial genomic DNA. By adding lysis buffer, the bacterial cells were broken down. The membrane retained the DNA in the extraction column, which was then eluted with an elution buffer. The amount of extracted DNA was assessed by spectrophotometric measurement at 260 nm (A260). The antimicrobial resistance genes (*bla*Z, *mec*A, *aac*A-*aph*D, *msr*A, *tet*K, *gyr*A, *grl*A, *dfr*G, and *cfr*) [27] and *agr* [21] were identified through polymerase chain reaction (PCR). The specific primers for each gene are given in Table-1 [21, 27]. This PCR mixture, with a total volume of 25 μL, included 1 μM each antimicrobial resistance gene primer, 2 μM *agr* primer, 2.5 μL 10 U Taq PCR buffer, 0.2 mM deoxyribonucleotide triphosphates (dNTPs), 2 mM MgCl_2_, and 1 U Taq DNA polymerase (Thermo-scientific, Germany). The PCR mixture underwent a thermal cycling process on the Flexcycler^2^ (Analytik Jena, Germany) consisting of an initial denaturation at 95°C for 5 min, 30 cycles with denaturation at 95°C for 30 s (Table-1), annealing at gene-specific temperatures for 30 s, extension at 72°C for 60 s, and a final extension at 72°C for 10 min. About 1.5% agarose gel electrophoresis was used to analyze the amplified products, which were then stained with SYBR Safe (Invitrogen, USA). The DNA bands were observed under ultraviolet light using the Invitrogen UVP Bioimaging system (USA).

**Table-1:** PCR primer for amplification of antimicrobial resistant genes and *agr* group.

Target gene	Primer (5′–3′)	PCR amplicon (bp)	Reference
Beta-lactams *bla*Z	CAGTTCACATGCCAAAGAG TACACTCTTGGCGGTTTC	772	[27]
*mec*A	AAAATCGATGGTAAAGGTTGGC AGTTCTGCAGTACCGGATTTGC	533	[27]
Aminoglycosides *aac*A*-aph*D	CAAGAGCAATAAGGGCATAC CAATAGTTTCAATAGGATAA	936	[27]
Fluoroquinolones *gyr*A	AATGAACAAGGTATGACACC TACGCGCTTCAGTATAACGC	223	[27]
*grl*A	ACTTGAAGATGTTTTAGGTGAT TTAGGAAATCTTGATGGCAA	459	[27]
Macrolides *msr*A	GGCACAATAAGAGTGTTTAAAGG AAGTTATATCATGAATAGATTGTCCTGTT	940	[27]
Tetracyclines *tet*K	TTATGGTGGTTGTAGCTAGAAA AAAGGGTTAGAAACTCTTGAAA	348	[27]
Folate pathway inhibitors *dfr*G	CCCAAGGACTGGGAATATG TCCTCATAATTCACTTCTGG	326	[27]
Phenicols *cfr*	TGAAGTATAAAGCAGGTTGGGAGTCA ACCATATAATTGACCACAAGCAGC	746	[27]
agr group			
pan	ATGCACATGGTGCACATGC	439	[21]
*agr* I	GTCACAAGTACTATAAGCTGCGAT	572	
*agr* II	TATTACTAATTGAAAAGTGCCATAGC	320	
*agr* III	GTAATGTAATAGCTTGTATAATAATACCCA	657	
*agr* IV	GCGATAATGCCGTAATACCCG		

PCR=Polymerase chain reaction, agr=Accessory gene regulator

### Statistical analysis

The data were compiled and analyzed using GraphPad Prism version 5 (La Jolla, CA, USA). The antimicrobial resistance phenotypes of *S. aureus* and CoNS are presented as percentages with 95% confidence intervals.

## Results

### Rabbit abscess

**Figure-1:**
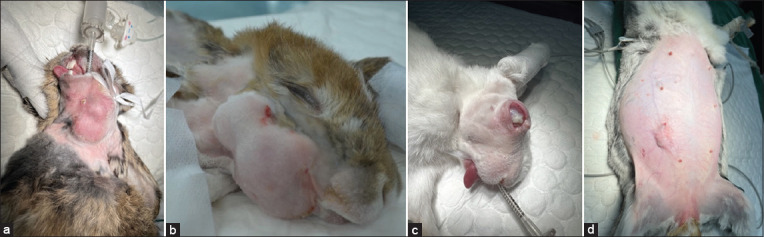
Illustrates abscesses in rabbits, encompassing (a) subcutaneous, (b) dental, (c) ocular, and (d) mammary gland manifestations.

Figure-1 depicts the various types of abscesses, such as the subcutaneous abscess (1A), dental abscess (1B), ocular abscess (1C), and mammary gland abscess (1D).

### Bacterial isolation and antimicrobial susceptibility testing

Among 67 samples collected from 67 rabbits, 19 isolates of *S. aureus* were identified from 9 abscesses (13.43%), whereas 37 isolates of CoNS were obtained from 18 abscesses (26.87%). Both *S. aureus* and CoNS were predominantly found in subcutaneous abscesses (Table-2). These isolates included 1 strain (5.26%) of MRSA and 22 strains (59.46%) of methicillin-resistant CoNS (MRCoNS).

**Table-2:** *Staphylococcus aureus* and coagulase-negative staphylococci (CoNS) isolated from clinical abscess.

Type of abscess specimen	No. of specimen collection (%)	*S. aureus*		CoNS	

		No. of specimen (%)	No. of isolated	No. of specimen (%)	No. of isolated
Subcutaneous	34 (50.75)	4 (5.97)	11	10 (14.93)	20
Dental	29 (43.28)	3 (4.48)	4	7 (10.45)	16
Ocular	2 (2.99)	1 (1.49)	3	0 (0)	0
Mammary gland	1 (1.49)	1 (1.49)	1	0 (0)	0
Uterine	1 (1.49)	0 (0)	0	1 (1.49)	1
Total	67 (100)	9 (13.43)	19	18 (26.67)	37

**Figure-2:**
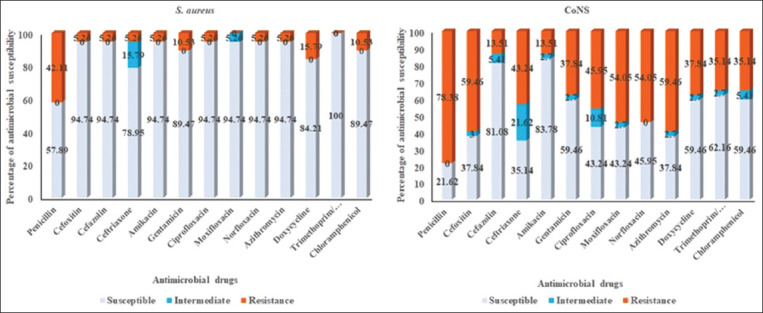
Antibiogram profile of Staphylococcus aureus and coagulase-negative staphylococci isolated from rabbits with clinical abscesses.

The susceptibility of *S. aureus* and CoNS isolates to antimicrobials was determined. About 42.11% of *S. aureus* and 78.38% of CoNS were identified as P-resitant (Figure-2). However, *S. aureus* isolates exhibited greater resistance to DO (15.79%) and C (10.53%), whereas CoNS had greater resistance to AZM (59.46%), MXF (54.05%), and NOR (54.05%). CoNS exhibited a higher resistance rate to a broader array of antimicrobial drugs compared to *S. aureus*. Fifty-nine-point four six percent of the CoNS isolates displayed MDR. The antimicrobial resistance of *S. aureus* and CoNS is presented in Tables-3 and 4.

**Table-3:** Pattern of antimicrobial resistance of *Staphylococcus aureus*.

Pattern of antimicrobials resistance of *Staphylococcus aureus*	Resistant gene	No. of resistance isolates (%)	*agr* group (No.; %)
Susceptible to all drugs	-	8 (42.11)	I (8; 42.11)
P	*bla*Z	5 (26.32)	I (3; 15.79), II (2; 10.53)
C	*cfr*	2 (10.53)	I (2; 10.53)
AK, CN	*aac*A*-aph*D	1 (5.26)	III (1; 5.26)
P, DO	*bla*Z, *tet*K	2 (10.53)	III (2; 10.53)
P, FOX, KZ, CRO, CN, CIP, MXF, NOR, AZM, DO	*bla*Z, *mec*A, *gyr*A, *grl*A, *msr*A	1 (5.26)	I (1; 5.26)

AK=Amikacin, AZM=Azithromycin, C=Chloramphenicol, CIP=Ciprofloxacin, CN=Gentamicin, CRO=Ceftriaxone, DO=Doxycycline, FOX=Cefoxitin, KZ=Cephazolin, MXF=Moxifloxacin, NOR=Norfloxacin, P*=*Penicillin

**Table-4:** Pattern of antimicrobial resistance of coagulase-negative staphylococci.

Pattern of antimicrobials resistance of coagulase-negative staphylococci	Resistant gene	No. of isolate
Susceptible to all drugs	-	4
P	-	1
P	*bla*Z	4
C	-	2
C	*cfr*	1
P, FOX	*mec*A	1
AK, AZM	*msr*A	1
P, FOX, MXF	*bla*Z, *mec*A	1
P, FOX, CRO, MXF	*bla*Z, *mec*A	1
P, FOX, MXF, NOR, AZM	*bla*Z, *mec*A, *gyr*A, *grl*A, *msr*A	1
P, AK, CN, CIP, NOR, AZM	*bla*Z, *aacA-aphD*, *gyr*A, *grl*A, *msr*A	1
P, AK, CN, CIP, AZM, SXT	*bla*Z, *aac*A*-aph*D, *gyr*A, *grl*A, *msr*A, *dfr*G	1
P, FOX, CRO, CN, NOR, AZM, C	*bla*Z, *mec*A, *aac*A*-aph*D, *gyr*A, *grl*A, *msr*A	1
P, FOX, CN, CIP, MXF, NOR, AZM	*bla*Z, *mec*A, *gyr*A, *grl*A, *msr*A	2
P, FOX, CRO, CN, CIP, MXF, NOR, AZM	*bla*Z, *mec*A, *aac*A*-aph*D, *gyr*A, *grl*A, *msr*A	1
P, FOX, CN, CIP, MXF, NOR, AZM, DO, C	*bla*Z, *mec*A, *aac*A*-aph*D, *gyr*A, *grl*A, *msr*A	1
P, FOX, CRO, CN, CIP, MXF, NOR, AZM, DO, SXT	*bla*Z, *mec*A, *aac*A*-aph*D, *gyr*A, *grl*A, *msr*A, *tet*K, *dfr*G	1
P, FOX, CRO, CN, CIP, MXF, NOR, AZM, DO, C	*bla*Z, *mec*A, *gyr*A, *grl*A, *msr*A, *tet*K	1
P, FOX, CRO, CIP, MXF, NOR, AZM, DO, SXT, C	*bla*Z, *mec*A, *gyr*A, *grl*A, *msr*A, *tet*K, *dfr*G	3
P, FOX, CRO, CIP, MXF, NOR, AZM, DO, SXT, C	*bla*Z, *mec*A, *gyr*A, *grl*A, *msr*A, *tet*K, *dfr*G, *cfr*	3
P, FOX, KZ, CRO, CN, MXF, NOR, AZM, DO, SXT	*bla*Z, *mec*A, *aac*A*-aph*D, *gyr*A, *grl*A, *msr*A, *tet*K, *dfr*G	1
P, FOX, KZ, CRO, AK, CN, MXF, NOR, AZM, DO, SXT	*bla*Z, *mec*A, *aac*A*-aph*D, *gyr*A, *grl*A, *msr*A, *tet*K, *dfr*G	1
P, FOX, KZ, CRO, CN, CIP, MXF, NOR, AZM, DO, SXT	*bla*Z, *mec*A, *aac*A*-aph*D, *gyr*A, *grl*A, *mrs*A, *tet*K, *dfr*G	1
P, FOX, KZ, CRO, AK, CN, CIP, MXF, NOR, AZM, DO, SXT	*bla*Z, *mec*A, *aac*A*-aph*D, *gyr*A, *grl*A, *msr*A, *tet*K, *dfr*G	1
P, FOX, KZ, CRO, CN, CIP, MXF, NOR, AZM, DO, SXT, C	*bla*Z, *mec*A, *aac*A*-aph*D, *gyr*A, *grl*A, *dfr*G	1

AK=Amikacin, AZM=Azithromycin, C=Chloramphenicol, CIP=Ciprofloxacin, CN=Gentamicin, CRO=Ceftriaxone, DO=Doxycycline, FOX=Cefoxitin, KZ=Cephazolin, MXF=Moxifloxacin, NOR=Norfloxacin, P*=*Penicillin, SXT=Trimethoprim/sulfamethoxazole

### Detection of antimicrobial resistance genes and *agr*

**Figure-3:**
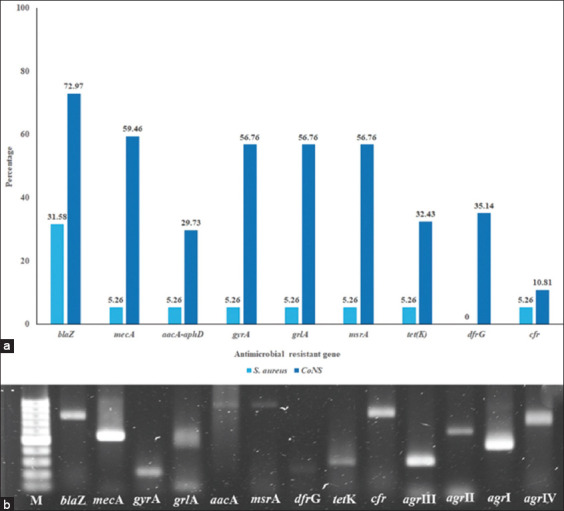
Antimicrobial resistance genes carried by *Staphylococcus aureus* and coagulase-negative staphylococci isolated from rabbits with clinical abscesses (a) and antimicrobial resistance genes product amplification by PCR (b). Lane M represents 100 bp ladder, lane 2 represents *bla*Z amplicon, lane 3 represents *mec*A amplicon, lane 4 represents *gyr*A amplicon, lane 5 represents *grl*A amplicon, lane 6 represents *aac*A*-aph*D amplicon, lane 7 represents *msr*A amplicon, lane 8 represents *dfr*G amplicon, lane 9 represents *tet*K amplicon, lane 10 represents *cfr* amplicon, lane 11 represents *agr* III amplicon, lane 12 represents *agr* II amplicon, lane 13 represents *agr* I amplicon, and lane 14 represents *agr* IV amplicon. PCR=Polymerase chain reaction, *agr*=Accessory gene regulator.

Antimicrobial resistance genes were detected in the *S. aureus* isolates. CoNS bore more antimicrobial resistance genes, among which *bla*Z was the predominant one linked to P resistance in *S. aureus*. CoNS showed a higher frequency of antimicrobial resistance genes such as *gyr*A, *grl*A, and *msr*A (Figure-3), while carrying more combinations of such genes than *S. aureus* (Table-4). Nineteen *S. aureus* isolates were analyzed, determining that 73.68% belonged to *agr* group I, whereas 15.79% and 10.53% belonged to *agr* groups III and II, respectively. (Table-3) In this study, *agr* group IV was not detected.

## Discussion

Clinical abscesses commonly harbor pathogenic staphylococci bacteria. Studies on staphylococci in companion animals have mostly focused on other species, with little data available on rabbits [9, 11–14]. About 13.43% of *S. aureus* and 26.87% of CoNS were diagnosed from clinical abscesses in rabbits (19 and 37 isolates, respectively) in our study. Subcutaneous abscesses were common in rabbits and were mostly caused by both *S. aureus* and CoNS [28]. Our study recorded a smaller proportion of *S. aureus* compared to 76% [28], 71% [29], and 19.1% [3]. The discrepancy seen in our study compared to previous research is due to the difference in rabbit populations, with ours consisting of individually raised animals and those in earlier studies being part of farm populations [3, 28, 29]. Although opportunistic, CoNS was frequently isolated from infected lesions in our study. The prevalence of CoNS in our study on rabbits falls within the reported range of 17.5%–63% in cats and dogs studies [5, 30]. CoNS attention is drawn due to their presence in sites of infection, high drug resistance rates and role as resistance gene reservoirs [1, 8, 31].

Antimicrobial resistance has gradually increased due to the overuse of antibiotics. Although less attention is given to it, the regulation of antibiotic use in companion animals is markedly different from that of food animal production systems [32]. Since 2004, numerous studies, including those by Goni *et al*. [9] and Vancraeynest *et al*. [11] have reported antimicrobial resistance in *S. aureus* isolated from rabbits, which varied according to the research’s time and location. While Vancraeynest *et al*. [9], Wang *et al*. [4], and Chai *et al*. [3] found low resistance rates (<25%). Attili *et al*. [29] identified high resistance rates with *S. aureus* strains being resistant to tetracycline (95.8%), clindamycin (93.8%), and erythromycin (93.8%).

About 5.26% of MRSA isolates in this study showed MDR. Antimicrobial resistance genes such as *tet*K, *msr*, *erm*, and *aac*(6’)-*aph*(2’) have earlier been detected in MRSA strains derived from rabbits [9, 28]. Tetracycline- and macrolide-resistant *S. aureus* isolates from our study carried the *tet*K and *msr*A genes, respectively. However, the isolates exhibiting aminoglycoside resistance in this study carried the *aac*A-*aph*D gene, whereas isolates from other studies carried the *aac*(6’)-*aph*(2’) gene [28]. Additionally, we also identified other resistance genes such as *bla*Z (P resistance), *gyr*A and *grl*A (quinolone resistance), and *cfr* (phenicol resistance).

The role of CoNS in causing disease in animals is still under debate in veterinary medicine. Previous research by Suepaul *et al*. [1], Elnageh *et al*. [30], and Michels *et al*. [33] indicates that animals with CoNS bacteria exhibit a wide range of antimicrobial resistance, making them potential repositories for resistance genes. The prevalence of CoNS in this study (26.87%) aligns with the range observed in companion animals (6%–63%) [5, 7, 30, 32] and is double that of *S. aureus* (13.43%). About 78.38% of CoNS in this study showed resistance to pencillin, 59.46% to AZM, 54.05% to MXF, and 54.05% to NOR, all due to the presence of antimicrobial resistance genes (*bla*Z, *msr*A, *gyr*A, and *grl*A). Surprisingly, the occurrence of MRCoNS was more frequent than *S. aureus*, even outnumbering reports in companion animals [5, 34]. Methicillin-resistant strains are known to exhibit heightened resistance across various antibiotic classes and pose a potential risk for transferring antimicrobial resistance genes between staphylococci, particularly those with higher pathogenicity, such as *S. aureus* [2, 34]. Our study overlooked the significance of the *mec*C gene, a critical factor in methicillin resistance in *Staphylococcus*, potentially limiting our understanding of antibiotic resistance mechanisms. Further study of this gene may lead to a more comprehensive understanding of resistance mechanisms and improved treatment outcomes. MDR strains are disproportionately represented among MRSA and CoNS isolates. The data in Table-4, along with the findings of Conner *et al*. [32], confirm the higher antimicrobial resistance prevalence in MRCoNS.

The *agr* is responsible for managing the expression of staphylococcal virulence factors and additional accessory genes. It significantly boosts the secretion of virulence factors at the expense of reducing those attached to the cell membrane. The correlation between clinical conditions, including enterotoxins diseases, infective endocarditis, toxic shock syndrome, and exfoliative diseases, and the four *agr* groups (*agr* I, II, III, and IV), has been emphasized by numerous studies [15]. US nosocomial MRSA isolates have been primarily *agr* II, while community-acquired strains predominantly carry the *agr* III genotype [18, 24, 35, 36]. Cafiso *et al*. [37] linked GISA and biofilm production to the *agr* II genotype.

Isolates with a positive *agr* system harbor more virulence genes than those with a negative *agr* system [38]. Isolates having the *agr* system were more vulnerable to antibiotics than those without it, with *agr* III strains being more susceptible than *agr* I strains to each tested antibiotic [23, 38]. In agreement with previous studies by Sakoulas *et al*. [15], Yoon *et al*. [17], Bibalan *et al*. [18], Javdan *et al*. [19], and Cheraghi *et al*. [20], *agr* I was the most common *agr* group found in our isolates. No *agr* IV isolates were found, indicating regional differences in *agr* group occurrence. *Agr* I was identified predominantly in samples from Brazil, Portugal, Hungary, and Berlin; *agr II* in isolates from Japan and North America; and *agr III* in European samples [36]. The distribution pattern of agricultural groups across diverse geographical regions reveals distinct populations of *S. aureus*. Monitoring drug resistance trends over time is essential for preserving antibacterial product efficacy. Veterinarians rely on antimicrobial susceptibility data to determine the most effective antibiotics for treating infections. Effective implementation of preventive measures and antimicrobial stewardship in veterinary and animal medicine are crucial for thwarting antimicrobial resistance [30, 34, 39].

## Conclusion

In rabbits, *S. aureus*, a *Staphylococcus* species, stands out for its heightened pathogenicity, causing clinical abscesses. CoNS is most commonly isolated from abscesses in studies. These bacteria carry multiple antimicrobial resistance genes, such as *bla*Z, *mec*A, *aac*A-*aph*D, *msr*A, *tet*K, *gyr*A, *grl*A, *dfr*G, and *cfr*, rendering them resistant to various antimicrobial drugs. The effectiveness of drugs against infections caused by these bacteria is restricted. Effective use of antibiotics in rabbits is necessary to prevent the propagation of antibiotic resistance. Educating rabbit owners to implement appropriate antimicrobial programs is essential in addressing this concern.

## Authors’ Contributions

SB and NI: Conceptualized and designed the study. NS: Carried out sample collection and animal care. NS, AJ, SS, TK, WT, NI, and SB: Conducted the study. NS, AJ, NI, and SB: Analyzed the data. SB: Provided reagents, materials, and analysis tools. NS, SS, NI, and SB: Wrote and edited the manuscript. All authors have read, reviewed, and approved the final manuscript.
